# Cardioprotective Effects of the Natural Antioxidant Epigallocatechin Gallate

**DOI:** 10.3390/antiox14121417

**Published:** 2025-11-27

**Authors:** Haiyang Li, Yuyang Zhang, Zhaoyang Hu, Jin Liu

**Affiliations:** 1 Department of Anesthesiology, West China Hospital, Sichuan University, Chengdu 610041, China; haiyangliapril@outlook.com (H.L.);; 2 Laboratory of Anesthesia and Critical Care Medicine, National-Local Joint Engineering Research Centre of Translational Medicine of Anesthesiology, West China Hospital, Sichuan University, Chengdu 610041, China

**Keywords:** epigallocatechin gallate, metabolic disorders, coronary artery disease, adverse cardiac remodeling, myocardial infarction, myocardial ischemia–reperfusion injury, cardiomyocyte injury, cardiac function

## Abstract

Cardiovascular health is essential for human survival, yet it remains threatened by injuries associated with metabolic disorders, myocardial infarction, ischemia–reperfusion, and even general anesthesia. The development of a safe and effective cardioprotective agent would be of considerable value in diverse clinical settings. Epigallocatechin gallate (EGCG), the principal constituent of catechins, has garnered considerable attention owing to its diverse health benefits. As a natural antioxidant and anti-inflammatory agent, it has been shown in numerous studies to exert pronounced cardioprotective effects. In preclinical studies, EGCG not only protects the coronary arteries but also attenuates adverse cardiac remodeling, prevents regulated cell death of cardiomyocytes, and enhances cardiac function. In addition, clinical studies have confirmed its beneficial effects on metabolic disorders, endothelial dysfunction, and adverse cardiac remodeling.

## 1. Introduction

Cardiac health is fundamental to overall human well-being, yet it is increasingly challenged by a variety of factors. Metabolic disorders, such as obesity, metabolic syndrome (MS), and type 2 diabetes mellitus (T2DM), have emerged as major health threats in the modern world [1], contributing to cardiovascular problems. Abnormalities in multiple metabolic components caused by these diseases impose persistent stress on the cardiovascular system, ultimately leading to coronary artery disease (CAD) and adverse cardiac remodeling [2]. Myocardial infarction (MI), the most severe clinical manifestation of CAD, affects approximately 3.8% of the global population under 60 years of age and up to 9.5% of those over 60 [3]. Another pressing issue associated with the rising incidence of MI is the lack of effective pharmacological interventions to prevent or treat myocardial ischemia–reperfusion injury (MIRI) during reperfusion therapy. In addition, with the widespread adoption of medical technology, the number of anesthesia cases has been rapidly increasing, rising annually by approximately 10% in China between 2015 and 2017 [4]. Consequently, the incidence of myocardial injury after non-cardiac surgery (MINS) is also expected to rise. This is defined as an elevation in cardiac troponin (cTn) concentrations within 30 days postoperatively, and is a frequent complication associated with increased postoperative mortality. Because most cases are asymptomatic, MINS is often overlooked [5].

Given the multiple threats to cardiac health, developing cardioprotective agents holds considerable clinical significance. Natural compounds, compared with synthetic substances, may be more suitable as therapeutic agents and play a pivotal role in drug development [6]. Tea has demonstrated a wide range of health benefits [7], for its rich content of polyphenolic compounds. Catechins are the main polyphenolic compounds in green tea (GT), and the unique polyphenolic compounds in other types of tea are mostly derived from their oxidation and fermentation processes [8]. EGCG, which represents 50–80% of the total catechins in GT, exhibits the strongest antioxidant capacity owing to its abundant hydroxyl groups [9]. As a natural anti-inflammatory and antioxidant substance, EGCG improves glucose and lipid metabolism [10], and exerts preventive and therapeutic effects in CAD [11,12] and diabetic cardiomyopathy (DCM) [2]. Furthermore, it demonstrates substantial potential in MI, MIRI [13], restrictive cardiomyopathy (RCM) [14], and heart failure (HF) [15], underscoring its broad cardioprotective effects. This review aims to summarize the cardioprotective mechanisms of EGCG and to evaluate its translational potential as a therapeutic antioxidant. We also discuss its potential adverse reactions and future research directions, emphasizing the significant impact of rational dosing regimens on outcomes.

## 2. Cardioprotective Effects

### 2.1. Coronary Artery Protection

**Figure 1:**
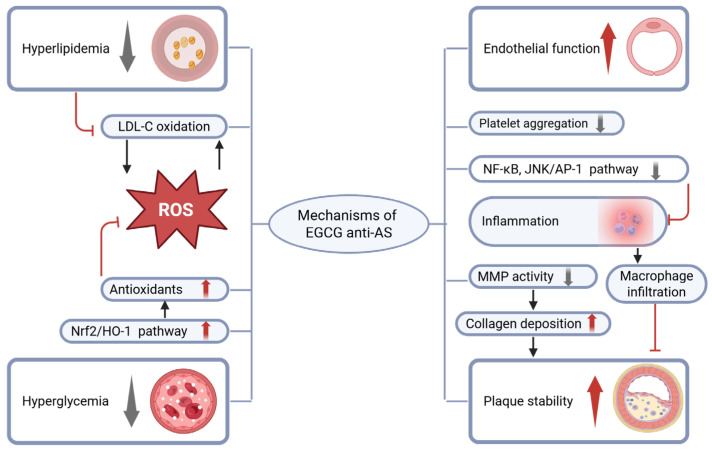
EGCG improves metabolic disorders, reducing hyperlipidemia and hyperglycemia. At the same time, EGCG activates the Nrf2/HO-1 pathway, increasing the levels of antioxidants. The increase in ROS and low-density lipoprotein levels leads to an increase in the oxidation of low-density lipoprotein, which then stimulates the production of more ROS and inflammatory factors by macrophages and endothelial cells, thereby exacerbating the endothelial dysfunction. EGCG also reduces the levels of ROS and low-density lipoprotein, improving endothelial function, and inhibits platelet aggregation, which is helpful for preventing thrombosis. In addition, EGCG inhibits macrophage infiltration in plaques by suppressing signaling pathways such as NF-kB and JNK/AP-1, inhibits the activity of matrix metalloproteinases, and increases the collagen fiber content in plaques, thereby enhancing plaque stability. The grey downward arrow in the labels indicates a decrease while the red upward arrow indicates an increase. The black arrows between the labels indicate a promoting effect while the red arrows represent an inhibitory effect.

Atherosclerosis (AS) is a chronic inflammatory vascular disorder primarily induced by hyperlipidemia, hyperglycemia, smoking, and other risk factors. The primary pathological mechanism involves endothelial dysfunction and vascular wall injury induced by harmful stimuli, which promote lipid deposition and plaque formation, ultimately leading to CAD [16]. Chronic inflammation is critically involved in the initiation and progression of numerous diseases [17]. MS is also recognized as an inflammatory disorder [18]. EGCG, with potent antioxidant and anti-inflammatory activities, may play an essential role in protecting against MS [10]. The Nrf2/HO-1 signaling pathway, a major regulator of intracellular defense against oxidative stress, is regarded as an ideal target for alleviating endothelial cell injury [19]. EGCG not only attenuates oxidative stress via activation of the Nrf2/HO-1 pathway but also suppresses inflammatory responses in AS [20], thereby conferring endothelial protection. EGCG also exhibits anti-platelet aggregation effects, which are beneficial for preventing thrombosis. Matrix metalloproteinases (MMPs), key enzymes involved in extracellular matrix remodeling, are critical determinants of plaque stability. Numerous studies have indicated that polyphenols enhance plaque stability by suppressing MMP gene expression and enzymatic activity [21,22]. The main anti-AS mechanisms of EGCG are illustrated in Figure 1.

#### 2.1.1. Improvement in Metabolic Disorders

Clinical studies have demonstrated that long-term oral administration of EGCG exerts therapeutic effects on lipid abnormality (LA) associated with obesity [23,24,25] and smoking [26]. A clinical trial demonstrated that oral EGCG also has the effect of lowering blood pressure in obese patients [27]. Although another clinical trial reported no lipid-lowering effect of oral EGCG at 400 mg twice daily in obese patients [28] while noting a reduction in blood pressure, a systematic review demonstrated that a daily oral intake of 107–856 mg of EGCG for 4–14 weeks significantly reduced serum low-density lipoprotein cholesterol (LDL-C) levels [29]. Many research studies have reported that EGCG has blood pressure-lowering effects [30], with nitric oxide playing a significant role in this process [31]. As a powerful antioxidant, EGCG protects nitric oxide from oxidation, increases its levels, and therefore causes vasodilation. In patients with T2DM, a meta-analysis revealed that supplementation with green tea extract (GTE) for more than 8 weeks at doses exceeding 800 mg/day significantly reduced serum triglyceride (TG) concentrations [32]. Two clinical trials in T2DM patients with LA or obesity reported that daily oral administration of 1500 mg of GTE (≈865 mg of EGCG) for 16 weeks did not significantly improve insulin resistance or LA compared with placebo. However, both studies observed modest improvements in insulin resistance after 16 weeks of GTE treatment compared with baseline [33,34]. This outcome may be attributed to the bidirectional exacerbation between T2DM and LA, which resulted in more severe conditions in the study population [35], rendering the EGCG dosage and treatment duration insufficient. Extending EGCG administration to two months resulted in observable improvements in LA among T2DM patients [36]. By contrast, administration of EGCG in gestational diabetes mellitus (GDM), a condition less severe than T2DM, improved maternal glycemic indicators and reduced neonatal complications [37]. Although the anti-diabetic effects of EGCG have been validated in numerous preclinical studies [38,39,40], they remain inconclusive in humans. Across observational studies, the association between tea consumption and T2DM incidence has been inconsistent. Overall, drinking tea appears to confer some protection against T2DM, but the evidence remains inconclusive and may be confounded by lifestyle and dietary factors [41]. Moreover, tea contains other components besides EGCG, and the amount of EGCG provided by tea consumption is relatively low, so the issue is still unclear at present. More clinical studies using EGCG alone are needed to verify its anti-diabetic effects. Although current clinical trials have not demonstrated the significant anti-diabetic efficacy of EGCG, they have reported improvements in LA among T2DM patients, reductions in blood pressure [36], and amelioration of arterial stiffness [42], all of which are beneficial for CAD. The clinical trial findings regarding the coronary protective effects of EGCG and its inhibition of adverse cardiac remodeling are summarized in Table 1.

#### 2.1.2. Improvement in Endothelial Dysfunction

The vascular endothelium is pivotal in regulating vascular homeostasis, and preserving its vasodilatory, anti-inflammatory, antithrombotic, and antiproliferative functions is essential for mitigating cardiovascular risk. Notably, the endothelial protective effects of EGCG have been confirmed in clinical trials involving patients with early AS or CAD [43,44]. All preclinical studies investigating the coronary protective effects and the inhibition of adverse cardiac remodeling by EGCG are summarized in Table 2. In vivo animal studies demonstrated that EGCG activated the Nrf2/HO-1 pathway to attenuate oxidative stress, inhibited lipid peroxide formation, suppressed NF-κB signaling, and limited the release of inflammatory mediators, thereby conferring endothelial protection in high-fat-diet-fed mice [47,48,49]. Furthermore, human primary T cells cultured in vitro and pretreated with EGCG exhibited marked reductions in AP-1 activity and inflammatory cytokine secretion upon stimulation [50]. Collectively, the potent antioxidant and anti-inflammatory properties of EGCG safeguard endothelial integrity, a mechanism critical for attenuating the progression of CAD.

#### 2.1.3. Prevention of Coronary Thrombosis

In vitro studies have demonstrated that EGCG suppresses platelet aggregation [55,57], thereby contributing to thrombosis prevention. Furthermore, as nearly 76% of fatal coronary thrombi are triggered by plaque rupture, maintaining plaque stability is of critical importance for patients with AS. In AS mouse models, EGCG exhibited anti-inflammatory, antioxidant, and lipid-lowering effects, and further suppressed MMP-2 and MMP-9 activity in plaque tissues, reduced macrophage infiltration, increased smooth muscle cell and collagen content, thickened the fibrous cap, and ultimately enhanced plaque stability [51,52].

### 2.2. Inhibition of Adverse Cardiac Remodeling

The reconstruction of the cardiac extracellular matrix requires a finely regulated balance. Moderate fibrotic repair preserves cardiac structural stability, whereas excessive collagen deposition impairs cardiac function. Adverse cardiac remodeling occurs in multiple pathologies, including post-MI [60], DCM, HF, RCM, and hypertrophic cardiomyopathy (HCM). Consequently, inhibiting adverse cardiac remodeling represents a critical therapeutic strategy and objective. Whether in animal experiments or clinical trials, EGCG has demonstrated the ability to inhibit adverse cardiac remodeling.

#### 2.2.1. Inhibition of Collagen Deposition

In vitro studies have demonstrated that EGCG suppresses the proliferation of cardiac fibroblasts (CFs) and collagen (COL) deposition, potentially by downregulating the NF-κB pathway to decrease connective tissue growth factor (CTGF) expression [53], or by inhibiting the JNK/AP-1 pathway to attenuate endoglin expression [56]. Beyond its actions on CFs, EGCG also inhibits endothelial-to-mesenchymal transition (EndMT) and calcium deposition in vascular smooth muscle cells by suppressing NF-κB signaling [58] and inactivating JunB [59]. In diabetic mouse models, EGCG improved glucose and lipid metabolism, reduced cardiomyocyte apoptosis, and decreased the extent of cardiac fibrosis [39,40]. Similarly, EGCG treatment reduced COL deposition in mouse models of HF [53,54].

#### 2.2.2. Inhibition of Amyloid Deposition

Transthyretin is the most common precursor protein of hereditary amyloidosis, and its cardiac deposition leads to RCM. In contrast to the hereditary form, no characteristic mutations in the transthyretin gene are detected in patients with wild-type transthyretin amyloid cardiomyopathy (wtATTR-CM), a condition predominantly affecting elderly individuals, and the underlying pathogenic mechanisms remain to be elucidated. In clinical trials, long-term oral administration of EGCG reduced left ventricular mass in patients with wtATTR-CM [14,45]. Imaging analyses further revealed that the reduction in left ventricular mass originated not from cardiomyocytes (CMs) but from the extracellular matrix [46]. These findings suggest that EGCG may exert therapeutic benefits in wtATTR-CM by modulating extracellular matrix remodeling rather than directly affecting CMs.

### 2.3. Prevention of Cardiomyocyte Injury

MI and MIRI expose CMs to severe oxidative stress and inflammatory response, which consequently result in cardiomyocyte injury, mitochondrial damage, pro-apoptotic factor release, and apoptosis [61,62]. EGCG exerts potent antioxidant, anti-inflammatory, and anti-apoptotic effects [63,64], showing great potential for application in MI and MIRI. Beyond apoptosis, other forms of regulated cell death (RCD) have also increasingly attracted attention in the context of MI and MIRI [65,66]. Autophagy constitutes a crucial metabolic pathway that supports cell survival under conditions of nutrient deprivation, hypoxia, or oxidative stress. Excessive autophagy, however, aggravates cellular dysfunction and induces RCD [67]. Preclinical studies have demonstrated that MI and MIRI suppress the PI3K/Akt signaling pathway [68] and induce excessive autophagy in CMs, thereby increasing RCD, whereas EGCG administration markedly reversed these pathological responses. A meta-analysis confirmed the cardioprotective effects of EGCG against MI and MIRI in small-animal models [13]. However, there are currently no clinical studies to validate the efficacy of EGCG in MI and MIRI, and all preclinical studies on its protective effects against cardiomyocyte injury in different diseases are summarized in Table 3.

#### 2.3.1. Alleviation of Oxidative Stress and Inflammatory Response

Although the global ischemic injury induced by hypothermic cardiac arrest during cardiopulmonary bypass (CPB) is less severe than that observed in MI or MIRI and typically does not result in irreversible cardiac dysfunction, CMs are nevertheless exposed to oxidative stress and inflammatory damage, leading to apoptosis and elevated plasma myocardial enzyme levels, a condition partially resembling MINS. In a large-animal model simulating CPB injury, supplementation of the cardioplegic solution with EGCG alleviated cardiomyocyte injury [69]. Subcutaneous injection of isoproterenol (IPR) to induce MI in mice resulted in LA, heightened oxidative stress and inflammation, and subsequent cardiomyocyte injury. EGCG administration, whether before or after MI induction, significantly improved LA, lowered the atherogenic index of plasma (AIP), and elevated antioxidant defenses—including enzymatic antioxidants such as superoxide dismutase (SOD) and catalase (CAT), as well as non-enzymatic antioxidants such as vitamin C (VC), vitamin E (VE), and ceruloplasmin (CER)—in mice. These effects collectively reduced reactive oxygen species (ROS) and malondialdehyde (MDA) levels, decreased TNF-α expression, and ultimately alleviated cardiomyocyte injury [63,70,71,72,73,74,75].

#### 2.3.2. Alleviation of Mitochondrial Dysfunction

Preserving mitochondrial structure and function not only ensures energy supply for cellular activities but also constitutes a fundamental mechanism for preventing the initiation of mitochondrial apoptosis and sustaining cell survival. Not only do pathological processes such as MI and MIRI lead to mitochondrial dysfunction, but some anesthetics also have adverse effects on mitochondrial function [87]. For patients who require anesthesia, if damage is inevitable, a drug that can protect mitochondrial function becomes particularly important. Beyond the classical roles of Bcl-2 family proteins and the pro-apoptotic factor cytochrome c, EGCG mitigates mitochondrial injury and its downstream consequences through additional molecular pathways. In MI rat models, the activity of tricarboxylic acid cycle enzymes and mitochondrial respiratory chain marker enzymes in CMs was markedly reduced, whereas EGCG pretreatment significantly attenuated these alterations and preserved mitochondrial function [72]. Intravenous administration of EGCG (10 mg/kg) 5 min before reperfusion significantly reduced plasma levels of mitochondrial DNA (mtDNA) and inflammatory cytokines—including TNF-α, IL-6, and IL-8—in MIRI mice. Moreover, plasma mtDNA levels positively correlated with TNF-α, IL-6, and IL-8, suggesting that mtDNA may function as a pro-inflammatory mediator [77]. OPA1 is a key regulator of inner mitochondrial membrane fusion and cristae maintenance. OMA1, a metalloendopeptidase, initiates OPA1 proteolysis, and its short autolytic form exhibits hydrolytic activity. EGCG directly interacted with OMA1 and strongly inhibited its self-cleavage, thereby attenuating OPA1 proteolysis and suppressing mitochondrial apoptosis [80].

#### 2.3.3. Activation of the Protective PI3K/Akt Pathway

The PI3K/Akt signaling pathway serves as an intrinsic regulatory mechanism that promotes cell survival under harmful external stimuli, engaging multiple downstream targets and influencing diverse cellular functions. Multiple studies have demonstrated that the cardioprotective effects of EGCG in MI and MIRI are mediated through activation of this pathway, whereas pharmacological inhibition of PI3K/Akt signaling abolishes the protective effects of EGCG [76,77,78].

#### 2.3.4. Inhibition of Regulated Cell Death

**Figure 2:**
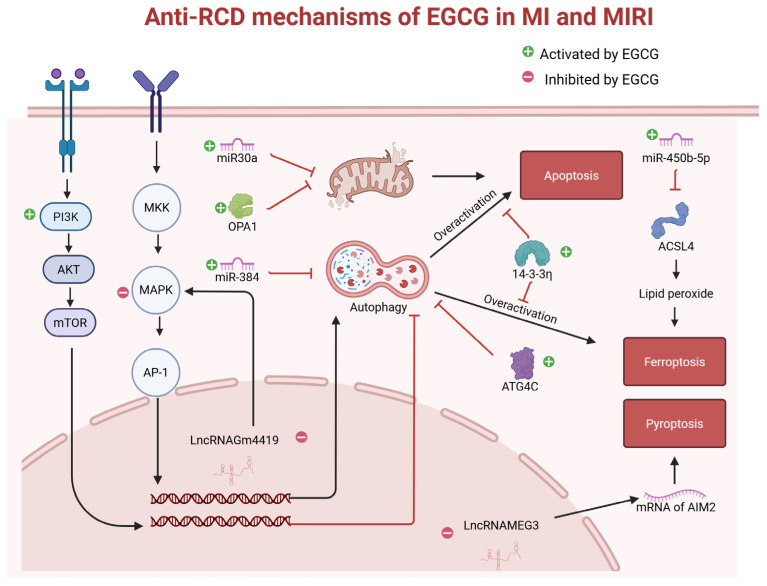
In MI and MIRI, cardiomyocytes often exhibit impaired PI3K signaling pathways and overactivation of MAPK pathways, leading to excessive autophagy and the induction of RCD. EGCG mitigates this phenomenon through the mediation of miR-384, LncRNAGm4419, 14-3-3η protein, and ATG4C protein. In addition, EGCG increases the expression level of miR-30a, inhibits the hydrolysis of OPA1, and thereby suppresses mitochondrial apoptosis. EGCG reduces ACSL4 protein expression by increasing the expression level of miR-450b-5p, thereby inhibiting ferroptosis, and it reduces the stability of AIM2 mRNA by lowering the expression level of LncRNAMEG3, thereby inhibiting pyroptosis. The black arrows between the labels indicate a promoting effect while the red arrows represent an inhibitory effect.

In addition to oxidative stress, inflammatory responses, and mitochondrial damage, MI and MIRI also induce excessive autophagy in CMs, leading to functional impairment and increased apoptosis. The inhibitory effects of EGCG on excessive autophagy and apoptosis have been confirmed in multiple studies [78,79,84]. miR-30a inhibits apoptosis by regulating gene expression, and EGCG treatment in MI mice upregulated miR-30a expression in CMs. Notably, miR-30a can also be transferred to neighboring CMs via exosomes, thereby exerting protective effects [81]. In recent years, beyond apoptosis, which has been extensively characterized, other forms of RCD—including ferroptosis and pyroptosis—have been recognized as critical contributors to MI and MIRI. Studies have shown that EGCG inhibits ferroptosis in CMs by upregulating 14-3-3η protein [86] and miR-450b-5p [82] in mice, and suppresses pyroptosis by downregulating long non-coding RNA (LncRNA) MEG3, which reduces the stability of AIM2 mRNA [85]. Collectively, through multiple pathways, EGCG suppresses RCD in CMs and enhances their survival, and the main anti-RCD mechanisms of EGCG in MI and MIRI are illustrated in Figure 2.

### 2.4. Preservation of Cardiac Function

Cardiac function is regulated by multiple factors. The inhibitory effects of EGCG on adverse remodeling, its preservation of mitochondrial function, and its suppression of RCD in MI and MIRI collectively contribute to the attenuation of cardiac dysfunction. Beyond MI and MIRI, in vivo mouse studies have validated the protective effects of long-term oral EGCG on cardiac function in aging, as well as manganese superoxide dismutase (Mn-SOD)-deficient and troponin-mutation-related RCM models [88,89,90]. Long-term oral administration of EGCG also enhanced tolerance to MIRI in T2DM mice [83], and spontaneously hypertensive rats (SHRs) [91], thereby mitigating cardiac dysfunction. Furthermore, EGCG directly interacts with cTn, modulates thin-filament function, increases ATP content, and enhances calcium transients, thereby improving contractile function at the single-cell level. All preclinical studies on the cardioprotective effects of EGCG on cardiac function are summarized in Table 4.

#### 2.4.1. Better Cardiac Structure

Adverse cardiac remodeling results in myocardial fibrosis and reduced compliance. The inhibitory effects of EGCG on adverse cardiac remodeling have been discussed in a previous section, including clinical trials evaluating its ability to inhibit transthyretin deposition; however, improvement in cardiac function was not the primary endpoint. Numerous in vivo mouse studies have demonstrated that long-term EGCG treatment ameliorated myocardial fibrosis and improved cardiac function in models of T2DM [93], HF [15,94,95,96,97], and post-MI [56,58].

#### 2.4.2. More Cardiomyocyte Survival

Increased cardiomyocyte survival supports cardiac function, and in vivo mouse studies demonstrated that emergency EGCG administration during MI and MIRI significantly reduced cardiomyocyte mortality and improved cardiac function [81,82,98,99]. In ex vivo experiments using the Langendorff isolated heart perfusion system (LIHPS) to maintain cardiac activity, supplementation of the perfusate with EGCG during the induction of MIRI [102,105] or CPB-related injury [101] reduced cardiomyocyte apoptosis and mitigated cardiac dysfunction. Although one ex vivo MIRI study [103] reported no improvement in cardiac function, another study [104] employing the same methodology but altering the timing of EGCG administration demonstrated significant functional improvement. The negative findings of the former study may be attributable to suboptimal timing of administration, as EGCG was not delivered during the reperfusion period.

#### 2.4.3. Better Cardiomyocyte Function

EGCG reduced mitochondrial injury in MI and MIRI mice and prevented downregulation of β1-adrenoceptors (β1-ARs) on cardiomyocyte membranes in HF mice [95], both of which favor enhanced cardiomyocyte contractile function. However, the effects of EGCG on β1-AR expression in cardiomyocyte membranes appear to be long-term, and whether it can elevate expression beyond baseline levels remains to be elucidated. EGCG increases calcium transients, enhancing the number of activated cross-bridges during excitation-contraction coupling, while also increasing ATP content to provide potential energy for more myosin, thereby improving cardiomyocyte contractile function. In addition, EGCG increases ATPase activity, accelerating the detachment of myosin and actin; reduces the increased calcium sensitivity caused by troponin mutations; and accelerates the detachment of troponin from calcium ions, thereby improving the diastolic function of cardiomyocytes. Even in the absence of injurious stimuli, EGCG treatment increased mitochondrial activity in mouse CMs and improved cardiac function [92,100]. In vitro studies have demonstrated that EGCG augments calcium transient peaks in CMs [100,106], thereby enhancing cardiomyocyte contractility. In addition, EGCG directly interacts with cTn, suggesting therapeutic potential for patients with troponin mutations. EGCG modulates troponin calcium sensitivity, accelerates calcium dissociation from troponin, facilitates sarcomere relaxation [88,107,109], restores excitation–contraction coupling [108], and increases maximal myosin ATPase activity [109], thereby enhancing thin-filament contractile and diastolic function.

## 3. Adverse Reactions of Epigallocatechin Gallate

In preclinical toxicology studies, administration of EGCG at 50 mg/kg intravenously (iv, once daily) for 10 days or 2000 mg/kg orally (po, once daily) for 10 days did not induce genotoxicity in mice [110]. Similarly, oral EGCG at 500 mg/kg daily for 13 weeks did not cause dermal irritation in rats [111], and dietary supplementation with 1400, 4200, or 14,000 mg/kg of EGCG during organogenesis was non-toxic to pregnant rats or their fetuses [112]. Oral EGCG reduces the bioavailability of iron [113], folic acid [114], and other drugs [115] by inhibiting their absorption. In vitro, hepatocytes exhibited damage upon exposure to 200 μM EGCG. In vivo, a single intraperitoneal injection of 100 mg/kg EGCG elevated plasma ALT levels in mice, and doses of 150 mg/kg caused death within 22 h [116]. Cai et al. reported that oral EGCG at 500 or 1000 mg/kg daily for 8 days induced mild cardiac fibrosis in mice [117]. Similarly, Rasheed et al. observed that intraperitoneal EGCG at 100 mg/kg daily for 4 days caused mild myocardial injury in diabetic mice [118]. In LIHPS experiments, low-dose EGCG (20 μM) enhanced cardiac contractility, whereas high-dose EGCG (50 μM) increased the incidence of arrhythmia and diastolic dysfunction [119].

In clinical studies, the most frequently reported adverse effects are hepatotoxicity and gastrointestinal disturbances, particularly when consumed on an empty stomach [120,121]. A few case reports have suggested that EGCG may cause severe liver injury [122]; however, given the very low incidence, current evidence is insufficient to establish a definitive causal relationship. In healthy volunteers, EGCG was shown to reduce folic acid bioavailability [123], although the doses and duration required to induce clinical folate deficiency remain unclear. Similarly, EGCG inhibits the uptake process of ticagrelor, while no potential drug–drug interaction risk was found based on microsomal data [124]. Given the inhibitory effects of EGCG on drug absorption mentioned earlier, to explore whether EGCG would affect the absorption of commonly used lipid-lowering drugs, Kim et al. conducted clinical studies and found that EGCG does mildly inhibit the absorption of rosuvastatin. However, after continuous administration of EGCG for about 10 days, this inhibitory effect was eliminated [125]. More interestingly, EGCG can reduce the metabolic rates of simvastatin [126] and rosuvastatin [127] and prolong their duration of action by inhibiting cytochrome P450 enzymes and organic anion transporting polypeptides. A daily oral dose of 800 mg of EGCG for 4 weeks was reported to be safe in healthy individuals [128]. According to the European Food Safety Authority, the average daily intake of EGCG from GT ranges from 90 to 300 mg/day, reaching up to 866 mg/day in heavy tea drinkers [129]. Thus, EGCG intake through tea consumption is considered safe. Large-scale cohort studies have reported no association between tea consumption during pregnancy and adverse outcomes [130]. However, there is limited direct evidence on the safety of EGCG in pregnant populations. Only one small-sample clinical study has shown that pregnant women with GDM taking 500 mg of EGCG orally every day for about 12 weeks is safe [37]. Subsequent clinical studies have shown that even higher oral doses of EGCG administered for 12 to 16 weeks (up to 865 mg/day) did not elicit significant adverse reactions, supporting its safety [24,33]. In a clinical trial, topical application of an EGCG spray (2574 μmol/L) three times daily for 2 weeks to treat severe radiotherapy-induced dermatitis revealed no EGCG-related adverse reactions [131].

Overall, the threshold doses of EGCG that induce serious adverse effects vary substantially depending on the administration route, treatment duration, and degree of oxidative stress in the subjects. Current clinical research primarily focuses on oral administration, typically at relatively low doses, while the safety profile of higher doses and alternative administration routes requires further investigation. Collectively, the available evidence indicates that EGCG is generally safe at dietary and clinically tested doses, but careful dose selection and population-specific evaluation remain essential for its therapeutic application. To elucidate our research results, we present a summary of effective and safe dosages of EGCG in different contexts in Table 5.

## 4. Conclusions and Future Perspectives

As a natural antioxidant and anti-inflammatory agent, long-term EGCG treatment improves glucose and lipid metabolism, prevents CAD progression, and alleviates adverse cardiac remodeling associated with T2DM, MI, HF, and related conditions. In acute injuries such as MI and MIRI, emergency EGCG administration reduces cardiomyocyte injury and suppresses RCD. The cardioprotective effects of EGCG have been validated in numerous cellular and small-animal studies. However, research in large-animal and comorbidity models remains limited, and clinical trials have primarily focused on metabolic outcomes, with few exploring EGCG in MI, MIRI, or CPB. Given its cardioprotective efficacy and favorable safety profile, further studies are warranted in large-animal models, comorbidity settings, and clinical trials assessing different EGCG dosages, administration routes, and timing strategies.

Particular attention should be paid to tailoring EGCG administration to disease-specific contexts. For instance, in the emergency management of MI and MIRI, intravenous administration is superior to oral delivery, as it circumvents the problem of low oral bioavailability, reduces the need for excessive oral dosing to achieve therapeutic concentrations, and minimizes the risk of adverse effects. By contrast, for chronic conditions such as T2DM, AS, and HF, oral administration is more practical, with dosing requirements—dependent on disease severity—differing substantially from those for intravenous therapy. In animal models, the safe oral dose may reach 500 mg/kg per day, whereas efficacy studies commonly employ ~50 mg/kg per day, equivalent to approximately 2500 mg/day in humans. Yet, the highest dose tested in current clinical trials does not exceed 1000 mg/day, and the safety and efficacy of higher oral doses remain to be clarified. For long-term oral administration, dividing the daily dose into three portions taken after meals is preferable, as this strategy reduces gastrointestinal irritation and minimizes fluctuations in plasma drug concentrations. Moreover, the timing of administration is critical. In MIRI, EGCG administration should precede reperfusion to counteract the surge of oxidative stress and inflammation during this phase.

Clinical investigations of EGCG can be extended to diverse clinical contexts. In designing dosing regimens, clinical applications should account for disease severity, the nature of injury (acute vs. chronic), and the practicality of administration. For example, to evaluate the preventive effect of EGCG on MINS, a relatively mild surgical complication, EGCG could be incorporated into the preoperative clear-liquid diet. To explore its therapeutic effect in MIRI, EGCG could be administered intravenously before or during reperfusion in patients with MI. For CPB, EGCG could be added to the cardioplegic solution to protect the arrested heart. In summary, dosing strategies for different administration routes must be carefully optimized, and the safety of these regimens should be rigorously monitored in human populations.

## Figures and Tables

**Table 1:** A summary of clinical trials on the cardioprotective effects of EGCG.

Study Population	Dose	Effect	References
88 obese male patients aged 40–65 years	400 mg of EGCG (po, bid) for 8 weeks	DBP ↓ NS between groups: HOMA-IR index, OGTT TG, TC, HDL-C, LDL-C	[28]
56 obese patients with hypertension	379 mg of GTE (po, qd) for 3 months	SBP, DBP ↓ HOMA-IR index, FBG ↓ TNF-α, CRP ↓ T-AOC ↑ TC, TG, LDL-C ↓ HDL-C ↑	[23]
102 women with central obesity	856.8 mg of EGCG (po, qd) for 12 weeks	TC, LDL-C ↓	[24]
30 obese patients	150 mg of EGCG (po, bid) for 8 weeks	Serum kisspeptin, TG ↓	[25]
30 obese patients	150 mg of EGCG (po, bid) for 8 weeks	SBP, DBP, MAP ↓	[27]
68 T2DM patients with obesity	1500 mg of GTE (po, qd) for 16 weeks (supplement to routine medication)	NS between groups: HOMA-IR index, FBG, HbA1c Within GTE group: Compared to baseline HbA1c ↓	[33]
77 T2DM patients with LA	500 mg of GTE (po, tid) for 16 weeks (supplement to routine medication)	NS between groups: HOMA-IR index, FBG, HbA1c TG, TC, HDL-C, LDL-C Within GTE group: Compared to baseline GLP-1 ↑ HOMA-IR ↓	[34]
326 pregnant women diagnosed with GDM during third trimester	500 mg of EGCG (po, qd) until full term	Improvement in maternal diabetes Cases of neonatal complications ↓	[37]
20 T2DM patients	400 mg of GTE (po, qd) for 12 weeks	Improved arterial stiffness	[42]
50 T2DM patients	300 mg of EGCG (po, bid) for 2 months (supplement to routine medication)	MAP, DBP ↓ TC, TG, AIP ↓ T-AOC ↑	[36]
120 South Indian male smokers	100 mL of GT (po, tid) for 1 year	Improved LA	[26]
52 patients with early AS	30 mL of olive oil with 280 mg of EGCG (po, qd) for 4 months (supplement to routine medication)	Endothelial function ↑	[43]
42 CAD patients	150 mg of EGCG (po, bid) for 2 weeks (supplement to routine medication)	Endothelial function ↑	[44]
19 patients with ATTR-CM	GT and/or GTE (exposure factors) for 1 year (supplement to routine medication)	Left ventricular mass ↓ TC, LDL-C ↓	[45]
25 male patients with wtATTR-CM	600 mg of EGCG (po, qd) for 1 year (supplement to routine medication)	Left ventricular mass ↓ Extracellular volume fraction ↓ TC ↓	[14,46]

The upward arrow indicates an increase of level, while the downward arrow indicates a decrease of level.

**Table 2:** A summary of preclinical studies on the protective effects of EGCG on atherosclerosis and adverse cardiac remodeling with different dosing regimens in various injury models.

Injury Models	Dosage Regimen	Results	References
Atherogenic diet for 45 days in male Wistar rats	EGCG 100 mg/kg (ip, qd) for the last 14 days	CRP, ESR ↓	[47]
High-fat diet for 15 weeks *P. gingivalis* (iv, tiw) for 3 weeks in ApoE-deficient mice	Drinking water with EGCG (0.2 g/L) for 7 weeks	AS areas ↓ CRP, IL-8, MCP-1 ↓ HO-1 ↑ oxidized LDL-C ↓	[48]
High-fat diet for 30 days in Wistar rats	EGCG 100 mg/kg (ip, qd) for 6/12 days	TC, TG, LDL-C ↓ HDL-C ↑ Antioxidants ↑ Lipid peroxidation ↓	[49]
High-fat diet for 16 weeks in ApoE-deficient mice	EGCG 10 mg/kg (ip, qd) for 16 weeks	TNF-α, IL-6, MCP-1, INF-γ ↓ EMMPRIN, MMP-2, MMP-9 ↓ Plaque stability ↑	[51]
High-fat diet for 6 weeks in ApoE-deficient mice	EGCG 10, 20, 40 mg/kg (po, qd) for 6 weeks	TC, TG, LDL-C, ↓ HDL-C ↑ VEGFA, MMP-2 ↓ SOD, Nrf2/HO-1 pathway ↑ ROS ↓	[52]
T2DM in db/db mice	Diet with EGCG (10 g/kg) for 10 weeks	FBG ↓ Plasma insulin ↑ Number of pancreatic islets ↑	[38]
NAM 100 mg/kg (ip) 20 min later STZ 55 mg/kg (ip) in male Wistar rats	After induction of DM, EGCG 2 mg/kg (po, qod) for 1 month	HOMA-IR index, FBG, HbA1c ↓ TG, TC, LDL-C, VLDL-C ↓ HDL-C ↑ SOD, CAT, GSH ↑ ROS ↓ IL- 1β, IL-6, TNF-α, ICAM-1, VCAM-1 ↓ cTnT, CK-MB, LDH, AST ↓ Histopathological injury ↓ Apoptosis ↓ Fibrosis area ↓	[39]
STZ 65 mg/kg (ip) in male SD rats	After induction of DM, EGCG 10, 20, 40 mg/kg (po, qd) for 12 weeks	FBG ↓ TG, TC, LDL-C ↓ HDL-C ↑ Fibrosis area, COL-I, COL-III ↓	[40]
AAC for 4 weeks in male SD rats	EGCG 25, 50 mg/kg (po, qd) for 4 weeks	NF-κB activation, CTGF ↓ Fibrosis area ↓	[53]
TAC for 4 weeks in male C57BL/6 mice	EGCG 20, 40, 80 mg/kg (po, qd) for 4 weeks	HW/BW, HW/TL, COL ↓ AKT/mTOR pathway ↓	[54]
COL (10 µg/mL) for 5 min with washed platelets from male SD rats	Preincubated with 1, 5, 10, 30, 50 μM EGCG for 3 min	Platelet aggregation ↓	[55]
100 nM Ang II for 24 h with CFs of adult rats	Preincubated with 1, 10 μM EGCG for 1 h	CF proliferation ↓ NF-κB, CTGF ↓ COL-Ⅰ, COL-Ⅲ ↓	[53]
10 nM Ang II for 4 h with CFs of adult rats	At the same time, EGCG 1, 10, 100 μM for 4 h	JNK/AP-1 ↓ Endoglin ↓ CF proliferation ↓	[56]
ADP 6.5 μM or COL 3.2 μg/mL for 6 min with blood samples from people taking anti-platelet drugs	EGCG 50, 100, 200 μM preincubated for 30 min	Platelet aggregation ↓	[57]
Human primary T cells incubated with P/I for 20 h	EGCG 10, 20 μM preincubated for 4 h	AP-1 binding activity ↓ IL-2, IL-4, INF-γ, TNF-α ↓	[50]
TGF-β2 10 ng/mL IL-1β 1 ng/mL for 24 h with HUVECs	After injury, EGCG 1, 5, 10 μM for 24 h	ROS ↓ NF-κB, SMAD pathways ↓ RhoA ↓ Cell migration ↓ EndMT ↓	[58]
10 mM β-GP and 3 mM CaCl2 with HASMCs	EGCG 20, 30 μM	JunB ↓ Osteogenic differentiation ↓ Mineral deposition ↓	[59]

The upward arrow indicates an increase of level, while the downward arrow indicates a decrease of level.

**Table 3:** A summary of preclinical studies on the protective effects of EGCG on cardiomyocyte injury with different dosing regimens in various injury models.

Injury Models	Dosage Regimen	Results	References
CPB Bypass-time for 90 min Reperfusion for 2 h in domestic piglets (10–15 kg)	Before CPB, EGCG 10 mg/kg (iv) After CPB, EGCG 10 mg/kg (iv)	CK ↓ Nitrosative and oxidative stress ↓ Inflammation ↓ Apoptosis ↓	[69]
IPR 100 mg/kg (sc, qd) for 2 days in male Wistar rats	After induction of MI, EGCG 10, 20, 30 mg/kg (po, qd) for 21 days	LDL-C, VLDL-C ↓ HDL-C ↑ AIP ↓ GSH, VC, VE, CER ↑ SOD, CAT ↑ MDA ↓ Mitochondrial damage ↓ Lysosomal enzymes ↓ CK, CK-MB, LDH, AST, ALT ↓ Histopathological injury ↓	[70,71,72,73,74,75]
IPR 100 mg/kg (sc, qd) for 2 days in male Wistar rats	Before induction of MI, EGCG 15 mg/kg (ip, qd) for 7 days	HW, HW/BW ↓ TC, TG, LDL-C ↓ HDL-C ↑ SOD, CAT ↑ MDA ↓ TNF-α ↓ CK-MB, LDH, ALT, ALP, cTnT ↓ DNA damage, Apoptosis ↓	[63]
LADO for 30 min Reperfusion for 2 h in male SD rats	5 min before reperfusion, EGCG 10 mg/kg (iv)	PI3K/AKT pathway ↑ p38, JNK ↓ Infarct size ↓	[76]
LADO for 30 min Reperfusion for 2 h in male Wistar rats	5 min before reperfusion, EGCG 10 mg/kg (iv)	PI3K/AKT pathway ↑ Plasma mtDNA, TNF-α, IL-6, IL-8 ↓ Incidence of ventricular arrhythmia ↓ Infarct size ↓	[77]
LADO for 30 min Reperfusion for 12 h in SD rats	30 min before ischemia, EGCG 10 mg/kg (iv)	PI3K/AKT pathway ↑ miR-384 ↑ Beclin-1, Excessive autophagy ↓ cTnI ↓ Infarct size ↓	[78]
LADO for 45 min Reperfusion for 3 h in male C57BL/6 mice	Before injury, EGCG 250 mg/kg (po, qd) for 10 days	LncRNA Gm4419 ↓ ERK1/2 ↓ Excessive autophagy ↓ Apoptosis ↓ Histopathological injury ↓ Infarct size ↓	[79]
H_2_O_2_ or HRI with MEFs or CMs of neonatal mice	Before injury, EGCG 20, 30, 40 μM for 1–3 h	Self-cleavage of OMA1 ↓ Proteolysis of OPA1 ↓ Mitochondrial function ↑ Mitochondrial morphology ↑ Apoptosis ↓	[80]
miR30a knockdown cells Hypoxia for 24 h	Exosomes from EGCG-treated CMs	miR30a ↑ Cell viability ↑	[81]
H_2_O_2_ 100 μM for 24 h with CMs of neonatal mice	EGCG (the dose is unknown)	LncRNA Gm4419 ↓ ERK1/2 ↓ Excessive autophagy ↓ Apoptosis ↓ Cell viability ↑ LDH ↓	[79]
HL-1 cells Hypoxia for 18 h	Before hypoxia, EGCG 5, 25 μM for 8 h	GSH, GPX4 ↑ ROS ↓ miR-450b-5p ↑ ACSL4, Ferroptosis ↓ Cell viability ↓	[82]
H9c2 cells in 30 mM glucose Hypoxia for 2 h Reoxygenation for 4 h	Before injury, EGCG 20 μM for 24 h	SIRT1 ↑ Mn-SOD ↑ MDA ↓ Apoptosis ↓ Cell viability ↑ LDH ↓	[83]
H9c2 cells Hypoxia for 6 h Reoxygenation for 12 h	Before injury, EGCG 6.25, 25 μM for 4 h	miR30a ↑ p53 ↓ Apoptosis ↓ CK-MB, LDH ↓ Cell viability ↑ ATP ↑	[81]
H9c2 cells Hypoxia for 6 h Reoxygenation for 12 h	Before injury, EGCG 25 μM for 4 h	PI3K/AKT pathway ↑ miR-384 ↑ Beclin-1, Excessive autophagy ↓ cTnI ↓ Cell viability ↑	[78]
H9c2 cells Hypoxia for 6 h Reoxygenation for 12 h	Before injury, EGCG 8 mg/L for 24 h	ROS ↓ ATG4C ↑ Excessive autophagy ↓ ATP ↑ Apoptosis ↓ Cell viability ↑	[84]
HL-1 cells Hypoxia for 2, 4, 8, 12 h Reoxygenation for 24 h	Before injury, 5, 10, 20, 40, 80, 100 μM of EGCG for 3 h	LncRNA MEG3 ↓ TAF15 in cytoplasm ↓ AIM2 mRNA stability ↓ Pyroptosis ↓ Cell death rate ↓ Cell viability ↑	[85]
H9c2 cells Hypoxia for 3 h Reoxygenation for 2 h	Before injury, EGCG 10 μM for 48 h	ROS, MDA ↓ 14-3-3η ↑ Excessive autophagy ↓ Ferroptosis, Apoptosis ↓ Cell viability ↑ LDH ↓	[86]

The upward arrow indicates an increase of level, while the downward arrow indicates a decrease of level.

**Table 4:** A summary of preclinical studies on the protective effects of EGCG on cardiac function with different dosing regimens in various injury models.

Injury Models	Dosage Regimen	Results	References
Healthy male Wistar rats	0.12 mg of EGCG (po, qd) for 28 days	Mitochondrial function ↑ ATP ↑ Cardiomyocyte mechanics ↑ Calcium transient ↑	[92]
Transgenic mice (cTnI-R193H)	EGCG 50 mg/kg (ip, qd) for 3 months	Diastolic function ↑	[88]
Senium C57BL/6 mice (16–18 months old)	At the age of 16 months, EGCG 50 mg/kg (ip, qd) for 8 weeks	Diastolic function ↑ HDAC1, HDAC3 ↓ cTnI ↑	[89]
Mn-SOD-deficient mice	At the age of 8 weeks, EGCG 10, 100 mg/L in drinking water for 8 weeks	Survival rate ↑ Cardiac dilatation ↓ Cardiac contraction ↑ Oxidative stress, Free fatty acids ↓ Telomerase activity ↓ Telomere length ↑	[90]
High-fat diet for 4 weeks STZ 30 mg/kg (ip) for 2 doses in 1 week in male SD rats	After induction of DM, EGCG 40, 80 mg/kg (po, qd) for 8 weeks	FBG ↓ CK-MB, cTnI ↓ Histopathological injury ↓ Autophagy, MMP2, MMP9 ↑ Fibrosis area, COL-Ⅰ, COL-Ⅲ ↓ LVSP, ±dp/dt max ↑ LVEDP ↓	[93]
TAC for 4 weeks in C57BL/6 mice	EGCG 10 mg/kg (ip, qd) for 4 weeks	Histopathological injury ↓ BNP ↓ Oxidative stress ↓ Inflammation ↓ Apoptosis ↓ LVEDD, LVESD ↓ LVEF ↑ TGF-β1/smad3 pathway ↓ COL-Ⅰ, COL-Ⅲ ↓	[15]
TAC for 12 weeks in C57BL/6 mice	After TAC, EGCG 50 mg/kg (ip, qd) for 12 weeks	Preventive effect on HF SERCA2a ↑	[94]
AAC for 4 weeks in rats	After AAC, EGCG (25, 50, 100 mg/kg/day) for 4 weeks	GRK2 ↓ β1-AR ↑ HW/BW, Posterior wall thickness ↓ LVSP, ±dp/dt max ↑ LVEDP ↓ Histopathological injury ↓	[95]
TAC for 12 weeks in mice	EGCG 50 mg/kg (ip, qd) for 12 weeks	HDAC5 ↓ Cardiac enlargement ↓ Cardiac function ↑	[96]
AAC for 16 weeks in male SD rats	8 weeks after AAC, EGCG 100 mg/kg (ip, qd) for 8 weeks	Cardiac function ↑ Myocardial hypertrophy, fibrosis ↓ Mitochondrial function ↑	[97]
LADO for 12 h in male SD rats	2 h before induction of MI, EGCG 10 mg/kg (iv)	miR30a levels ↑ CK-MB, cTnI ↓ Histopathological injury ↓ Excessive autophagy ↓ Apoptosis ↓ LVEF, LVSP, ±dp/dt max ↑ LVEDP ↓	[81]
LADO for 18 h in C57BL/6 mice	30 min before induction of MI, EGCG 5, 10, 20 mg/kg (iv)	SOD ↑ MDA ↓ miR-450b-5p ↑ ACSL4, Ferroptosis ↓ LVEDD, LVESD ↓ LVEF, FS ↑	[82]
LADO for 4 weeks in C57BL/6 mice	After induction of MI, EGCG 50 mg/kg (po, qd) for 4 weeks	CK-MB, LDH ↓ Histopathological injury ↓ LncRNA MEG3 ↓ Pyroptosis ↓ Cell death rate ↓ Infract size ↓ LVEF ↑	[85]
LADO for 14 days in adult Wistar rats	After induction of MI, EGCG 50 mg/kg (po, qd) for 14 days	Endoglin ↓ HW/BW, Fibrosis area ↓ LVEDD, LVESD ↓ MAP, FS ↑	[56]
LADO for 4 weeks in C57BL/6 mice	After induction of MI, EGCG 50 mg/kg (po, qd) for 1 week	1 week after MI: Snail (EndMT marker) ↓ MMP-2, MMP-9 ↓ COL-I, COL-III ↓ 4 weeks after MI: Apoptosis ↓ Infract size ↓ Fibrosis area ↓ Capillary density ↑ LVEF ↑	[58]
LADO for 30 min Reperfusion for 2 h in SD rats with DM	Before injury, EGCG 100 mg/kg (po, qd) for 2 weeks	SIRT1 ↑ Mn-SOD ↑ MDA ↓ LDH ↓ Apoptosis ↓ Infarct size ↓ Fibrosis area ↓ LVSP, ±dp/dt max ↑	[83]
LADO for 30 min Reperfusion for 2 h In male SD rats	10 min before reperfusion, EGCG 10 mg/kg (iv)	PI3K/AKT pathway ↑ Excessive autophagy ↓ CK-MB, LDH ↓ Nitric oxide ↑ Apoptosis ↓ Infarct size ↓ LVSP, ±dp/dt max ↑ LVEDP ↓	[98]
LADO for 30 min Reperfusion for 12 h in SD rats	30 min before ischemia, EGCG 10, 20 mg/kg (iv)	miR30a ↑ p53 ↓ Apoptosis ↓ CK-MB, LDH ↓ Histopathological injury ↓ ATP ↑ LVEF, LVSP, ±dp/dt max ↑ LVEDP ↓	[99]
LADO for 60 min Reperfusion for 2 h in C57BL/6 mice	Before injury, EGCG 20 mg/kg (po, qd) for 6 weeks	MDA ↓ Ferroptosis ↓ CK-MB, LDH ↓ Histopathological injury ↓ Infarct size ↓ LVEF ↑	[86]
LIHPS for hearts of male Wistar rats	EGCG 1, 4 μM in perfusate	LVSP, ±dp/dt max ↑	[100]
LIHPS for hearts of Chinchilla rabbits Cardioplegia for 90 min Reperfusion for 1 h	At the same time of cardioplegia, EGCG 20 μM in cardioplegic solutions for 90 min	Nitrosative and oxidative stress ↓ Apoptosis ↓ ATP ↑ LVSP ↑	[101]
LIHPS for hearts of male SHR Ischemia for 30 min Reperfusion for 2 h	Before injury, EGCG 200 mg/kg (po, qd) for 3 weeks	Coronary flow ↑ Infarct size ↓ LVDP ↑ LVEDP ↓	[91]
LIHPS for hearts of guinea pigs Ischemia for 40 min Reperfusion for 40 min	4 min before injury, EGCG 30 μM in perfusate	Mitochondrial Ca^2+^ elevation ↓ Apoptosis ↓ ATP ↑ LVEDP ↓	[102]
LIHPS for hearts of male Wistar rats Ischemia for 30 min Reperfusion for 2 h	10 min before ischemia, EGCG 1, 10 μM in perfusate for 40 min	Infarct size ↓ LVDP, ±dp/dt max NS Mitochondrial KATP activity ↑	[103]
LIHPS for hearts of male Wistar rats Ischemia for 30 min Reperfusion for 2 h	5 min before reperfusion, EGCG 1, 10 μM in perfusate for 35 min	Infarct size ↓ LVDP, ±dp/dt max ↑	[104]
LIHPS for hearts of male SD rats Ischemia for 20 min Reperfusion for 2 h	10 min before injury, EGCG 5 μM in perfusate for 130 min	Mn-SOD, Cu/Zn-SOD ↑ Lipid peroxides ↓ Apoptosis ↓ Infarct size ↓ LVDP, ±dp/dt max ↑ LVEDP ↓	[105]
CMs of adult rats	EGCG 2.5, 5 μM	Calcium transient ↑ FS ↑	[100]
CMs of C57BL/6 mice	EGCG 10 nM-100 μM	Calcium transient ↑	[106]
Human cTn subunits with cTnT-Δ160E mutation	EGCG 3 μM	Bind to the C-lobe of cTnC Binding of cTnI to cTnC ↑ Ca^2+^ sensitivity in myofilaments ↓	[107]
CMs of transgenic mice (cTnI-R193H)	EGCG 5 μM	Ca^2+^ decay, Sarcomere relaxation ↑	[88]
cTnT with mutations associated with HCM	EGCG 100 μM	Restore the coupling between Ca^2+^ and cTnT	[108]
Reconstituted TF with cTnC-G34S or cTnI-D127Y mutations	EGCG 20 μM	Aggregation and elongation of TF ↑ Maximal myosin-S1-ATPase activity ↑ Ca^2+^ sensitivity in myofilaments ↓	[109]

The upward arrow indicates an increase of level, while the downward arrow indicates a decrease of level.

**Table 5:** A summary of effective and safe dosages of EGCG in different contexts.

Dosage Regimens of EGCG	Benefits	Adverse Reactions
150 mg (po, bid) for 2 weeks	Endothelial function ↑ in CAD patients	Not found
150 mg (po, bid) for 8 weeks	TG ↓ SBP, DBP, MAP ↓ in obese patients	Not found
400 mg (po, qd) for 12 weeks	Arterial stiffness ↓ in T2DM patients	Not found
500 mg (po, qd) for about 12 weeks	Improvement in maternal diabetes Cases of neonatal complications ↓	Not found
300 mg (po, bid) for 2 months	MAP, DBP ↓ TC, TG, AIP ↓ in T2DM patients	Not found
600 mg (po, qd) for 1 year	LV extracellular mass ↓ in wtATTR-CM patients	Not found
856.8 mg (po, qd) for 12 weeks	TC, LDL-C ↓ in obese patients	Not found
10 mg/kg (po, qd) for 3 weeks	Cardiomyocyte injury ↓ in MI mice	Not found
10 mg/kg (po, qd) for 12 weeks	FBG ↓ Fibrosis area ↓ in DM mice	Not found
20 mg/kg (po, qd) for 4 weeks	Fibrosis area ↓ in HF mice	Not found
500 mg/kg (po, qd) for 8 days	Not applicable	Mild myocardial fibrosis in mice
10 mg/kg (ip, qd) for 4 weeks	Fibrosis area ↓ Cardiac function ↑ in HF mice	Not found
10 mg/kg (ip, qd) for 16 weeks	Plaque stability ↑ in AS mice	Not found
15 mg/kg (ip, qd) for 1 week before MI	Cardiomyocyte injury↓ in MI mice	Not found
100 mg/kg (ip, qd) for 1 day	Not applicable	ALT ↑ in mice
10 mg/kg (iv) for 1 dosage	Cardiomyocyte injury ↓ in MIRI mice	Not found
10 mg/kg (iv) for 2 dosages	Cardiomyocyte injury ↓ in CPB piglets	Not found
10 μM in perfusate of isolated hearts	Cardiomyocyte injury ↓ Cardiac function ↑ in MIRI mice	Not found
20 μM in cardioplegic solutions of isolated hearts	Cardiomyocyte injury ↓ Cardiac function ↑ in CPB rabbits	Not found
50 μM in perfusate of isolated hearts	Not applicable	Cardiac function ↓ in mice

The upward arrow indicates an increase of level, while the downward arrow indicates a decrease of level.

## Data Availability

Not applicable.

## Abbreviations

The following abbreviations are used in this manuscript:

ATTR-CM	Amyloidotic transthyretin cardiomyopathy
AS	Atherosclerosis
ApoE	Apolipoprotein E
AAC	Abdominal aortic constriction
Ang II	Angiotensin II
AIP	Atherogenic index of plasma
ALP	Alkaline phosphatase
AST	Aspartate aminotransferase
ALT	Alanine transaminase
ATG4C	Autophagy-related 4C
BW	Body weight
BNP	Brain natriuretic peptide
β-GP	β-Glucopyranosyl phosphate
β1-AR	β1-adrenoceptor
CAD	Coronary artery disease
CMs	Cardiomyocytes
CFs	Cardiac fibroblasts
CPB	Cardiopulmonary bypass
CAT	Catalase
CER	Ceruloplasmin
CRP	C-reactive protein
CTGF	Connective tissue growth factor
COL	Collagen
CK	Creatine kinase
CK-MB	Creatine kinase-MB
CO	Cardiac output
Cu/Zn-SOD	Cu, Zn-Superoxide dismutase
cTn	Cardiac troponin
cTnC	Cardiac troponin C
cTnT	Cardiac troponin T
cTnI	Cardiac troponin I
cTnI-R193H	Cardiac troponin I arginine 193 to histidine mutation
cTnT-Δ160E	Cardiac troponin T with glutamic acid deletion at position 160
cTnC-G34S	Cardiac troponin C glycine 34 to serine mutation
cTnI-D127Y	Cardiac troponin I aspartate 127 to tyrosine mutation
DM	Diabetic mellitus
DCM	Diabetic cardiomyopathy
DBP	Diastolic blood pressure
ESR	Erythrocyte sedimentation rate
EMMPRIN	Extracellular matrix metalloproteinase inducer
EndMT	Endothelial-to-mesenchymal transition
EGCG	Epigallocatechin gallate
FBG	Fasting blood glucose
FS	Fractional shortening
GT	Green tea
GTE	Green tea extract
GDM	Gestational diabetes mellitus
GLP-1	Glucagon-like peptide 1
GPX4	Glutathione peroxidase 4
GSH	Glutathione
GRK2	G protein-coupled receptor kinase 2
HCM	Hypertrophic cardiomyopathy
HF	Heart failure
HUVECs	Human umbilical vein endothelial cells
HASMCs	Human aortic smooth muscle cells
HRI	Hypoxia-reoxygenation injury
HDL-C	High-density lipoprotein cholesterol
HOMA-IR	Homeostasis model assessment of insulin resistance
HbA1C	Hemoglobin A1C
HW	Heart weight
HDAC	Histone deacetylase
IPR	Isoproterenol
ICAM-1	Intercellular adhesion molecule-1
KATP	ATP-sensitive potassium channels
LADO	Left anterior descending artery occlusion
LMCAO	Left main coronary artery occlusion
LIHPS	Langendorff isolated heart perfusion system
LA	Lipid abnormality
LDL-C	Low-density lipoprotein cholesterol
LDH	Lactate dehydrogenase
LVEF	Left ventricular ejection fraction
LVEDD	Left ventricular end-diastolic dimension
LVESD	Left ventricular end-systolic dimension
LVSP	Left ventricular systolic pressure
LVEDP	Left ventricular end-diastolic pressure
LVDP	Left ventricular developed pressure
LncRNA	Long non-coding RNA
MS	Metabolic syndrome
MI	Myocardial infarction
MIRI	Myocardial ischemia–reperfusion injury
MINS	Myocardial injury after non-cardiac surgery
MEFs	Mouse embryonic fibroblasts
Mn-SOD	Manganese superoxide dismutase
MDA	Malondialdehyde
MPO	Myeloperoxidase
MCP-1	Monocyte chemoattractant protein-1
MMPs	Matrix metalloproteinases
MAP	Mean arterial pressure
mtDNA	Mitochondrial DNA
NAM	Nicotinamide
NS	Not Significant
OGTT	Oral glucose tolerance test
P/I	Phorbol 12-myristate 13-acetate and ionomycin
*P. gingivalis*	Porphyromonas gingivalis
RCM	Restrictive cardiomyopathy
ROS	Reactive oxygen species
RCD	Regulated cell death
SD	Sprague-Dawley
SHR	Spontaneously hypertensive rats
STZ	Streptozotocin
SOD	Superoxide dismutase
SIRT1	Silent information regulator 1
SERCA2a	Sarcoplasmic reticulum Ca-ATPase
SBP	Systolic blood pressure
TF	Thin filament
TAC	Transverse aortic constriction
TC	Total cholesterol
TG	Triglyceride
T-AOC	Total antioxidant capacity
TL	Tibia length
TXA2	Thromboxane A2
T2DM	Type 2 diabetes mellitus
VLDL-C	Very low-density lipoprotein cholesterol
VC	Vitamin C
VE	Vitamin E
VEGFA	Vascular endothelial growth factor
VCAM-1	Vascular cell adhesion molecule-1
wtATTR-CM	Wild-type transthyretin amyloid cardiomyopathy
±dp/dt max	Maximal left ventricular pressure variation rate
